# Increased microvascular flow and foot sensation with mild continuous external compression

**DOI:** 10.1002/phy2.157

**Published:** 2013-12-19

**Authors:** Armando Rosales‐Velderrain, Michael Padilla, Charles H. Choe, Alan R. Hargens

**Affiliations:** 1 Department of Orthopaedic Surgery, University of California San Diego, San Diego, California

**Keywords:** Blood flow, compression, lower limb, Pneumatic, sensation

## Abstract

Intermittent pneumatic compression of the calf and foot increases inflow to the popliteal artery and skin. We hypothesize that mild, continuous pneumatic compression of the lower extremities of type 2 diabetic patients increases microvascular blood flow to skin (SBF) and muscle (MBF) and improves sensation in feet. Data were collected on 19 healthy volunteers and 16 type 2 diabetic patients. Baseline values of SBF, MBF, and foot sensation were recorded in one leg. The lower extremity was then subjected to 30 mmHg of continuous external air pressure for 30 min, whereas SBF and MBF were continuously monitored. Sensation was reassessed after pressure was released. During 30 mmHg continuous external compression, the healthy control group significantly increased MBF by 39.8% (*P* < 0.01). Sensation of the foot in this group improved significantly by 49.8% (*P* < 0.01). In the diabetic group, there was a significant increase in MBF of 17.7% (*P* = 0.03). Also sensation improved statistically by 40.2% (*P* = 0.03). Importantly and counterintuitively, MBF and foot sensation both increase after 30 min of leg compression at 30 mmHg. Therefore, mild, continuous pneumatic compression may be a new approach for treating diabetic patients with compromised leg perfusion and sensation.

## Abstract (precis)

Leg blood flow and foot sensation after external compression.

## Introduction

Therapies utilizing external compression of the leg are employed to prevent deep venous thrombosis, decrease lower extremity edema, manage chronic venous insufficiency, and increase healing in the treatment of venous stasis ulcers (van Bemmelen et al. 1994; Eze et al. 1996; Morris and Woodcock, 2004a; Trent et al. 2005). Moreover, the limbs of diabetic patients have decreased capillary density, reduced arterial inflow, and poor microvascular flow (Rizzoni and Rosei 2009). Poor perfusion limits wound healing and tissue viability, especially in patients with peripheral vascular disease and diabetes. Diabetic polyneuropathy (DPN) is present in 66% of type 1 and in 59% of type 2 diabetic patients (Dyck et al. 1993), with sensorimotor polyneuropathy the most common form (Kles and Vinik 2006). Vascular changes in DPN include increased capillary permeability and vasoconstriction, as well as a decreased blood flow, causing motor and sensory nerve fiber degeneration (Kles and Vinik 2006). Alterations in the microcirculation involve degeneration of the small arteries, arterioles, capillaries, and postcapillary venules (Mulvany and Aalkjaer 1990). Therefore, patients are at a higher risk of nontraumatic foot injuries and toe amputations (Kles and Vinik 2006). Moreover, van Bemmelen et al. (1994) report increased popliteal artery flow in healthy and diabetic subjects after intermittent calf compression for 2 sec with pressures ranging from 20 to 120 mmHg. Delis et al. (2000) detect increased popliteal artery flow in claudicant patients after 120 mmHg of intermittent pneumatic foot compression.

In other related studies, intermittent pneumatic compression (IPC) at high pressures of 120 mmHg produces postischemic elevations of skin perfusion and popliteal artery inflow (Eze et al. 1996, 1998; Trent et al. 2005). Also, cyclic IPC of the limb at 120 mmHg reduces venous pressure and, thus, increases the arteriovenous pressure gradient, thereby increasing blood flow (Labropoulos et al. 2005). Compression durations of 10 sec at 60 mmHg with moderate inflation rates in supine patients effectively increase flow velocity in the femoral artery (Morris and Woodcock 2002) meaning that systems do not necessarily require uncomfortable, high levels of compression, and rapid inflation periods to be efficacious. An optimal therapy for individual patients with peripheral vascular disease should increase microvascular flow in the limb for the longest period possible. Multiple mechanisms that cause arteriolar dilation have been reported as responsible for this blood flow increase, such as the myogenic response or vasodilator substance release (Bayliss 1902; Reneman et al. 1980; Mayrovitz and Larsen 1997).

However, to date, no study has documented the effect of continuous, low‐level pneumatic compression on microvascular flow and foot sensation in diabetic patients. The objective of our study is to evaluate foot sensation, skin and muscle microvascular flows over the anterior compartment of legs of diabetic patients and healthy volunteers during mild continuous pneumatic compression. We hypothesized that mild, continuous pneumatic compression of legs of diabetic patients increases microvascular blood flow to skin (SBF) and muscle (MBF) as well as improves foot sensation.

## Materials and Methods

Data were collected for 19 control healthy subjects (12 males, 7 females, 37.1 ± 13.8 years [mean ± SD]) and 16 patients (10 males, 6 females, age 48.2 ± 13.4 years [mean ± SD]) with a diagnosis of diabetes mellitus type 2. Subjects included in the diabetic group were previously diagnosed by a physician. The subjects' characteristics of age, weight, body mass index (BMI), and gender in our two volunteer groups are summarized in Table 1. Microvascular SBF and MBF were measured with a photoplethysmography device (PPG) (Morris and Woodcock 2002). The device was custom build by the University of California, San Diego Physics Department Electronics Shop. The PPG probe has two different light sources, a green light‐emitting diode (LED) with a 526‐nm wavelength that penetrates 3 mm into the skin tissue, and a near‐infrared LED with a wavelength of 805 nm that penetrates 13 mm corresponding to muscle tissue (Mateus and Hargens 2012). After light scatters around each layer, two light sensors in the probe detect the free light. Also, from this cohort of subjects, we collected sensation data using Semmes‐Weinstein monofilaments in 14 of the controls and 13 diabetic volunteers (Bell‐Krotoski and Tomancik 1987; Kles and Vinik 2006; Feng et al. 2009).

**Table 1.** Summary of baseline characteristics for normal controls and diabetic patients.

Characteristic	Group I, control (*N* = 19)*	Group II, diabetics (*N* = 16)*
Age (years)	37.1 ± 13.7	48.2 ± 13.4
Males	12	10
Females	7	6
Weight (kg)	83.2 ± 16.9	88.7 ± 17.3
Height (m)	1.76 ± 0.09	1.78 ± 0.13
BMI (kg/m²)	26.6 ± 4.26	28.1 ± 4.65
Glycosylated hemoglobin (%)	N/A	7.72 ± 1.75

* Values are given in mean ± SD.

In the diabetic group the most recent value in the charts of glycosylated hemoglobin (A1C) was recorded (Table 1). It should be noted here that the majority of the diabetic patients were prescribed a pharmacologic regimen by their physicians that included some type of statin in addition to a glucose control treatment. Furthermore, many of the diabetic volunteers were hypertensive for which they were prescribed antihypertensive medication.

After approval from our Institutional Review Board, subjects were recruited from November 2009 to November 2010 from the orthopedic clinic and through flyers. Subjects were informed of the project and agreed to participate. They signed an informed consent and were assigned to one of two groups: healthy volunteers or diabetic type 2 subjects. Skin and muscle microvascular blood flows were measured by PPG (Zhang et al. 2001a,b; Morris and Woodcock, 2004b).

Prior to pressurization, baseline skin and MBFs levels as well as sensation at the first web space of the foot (deep peroneal nerve distribution) were obtained in supine position. Skin sensibility was measured by Semmes‐Weinstein monofilaments (Bell‐Krotoski and Tomancik 1987; Kamei et al. 2005; Feng et al. 2009). Sensation measurements were taken while the patient was blinded, measurements started with the thicker monofilaments moving down until the last thinnest monofilament the patient was able to feel. During this process the patient was asked to notify the examiner each time they felt the monofilament in the first web space of their foot.

**Figure 1.**
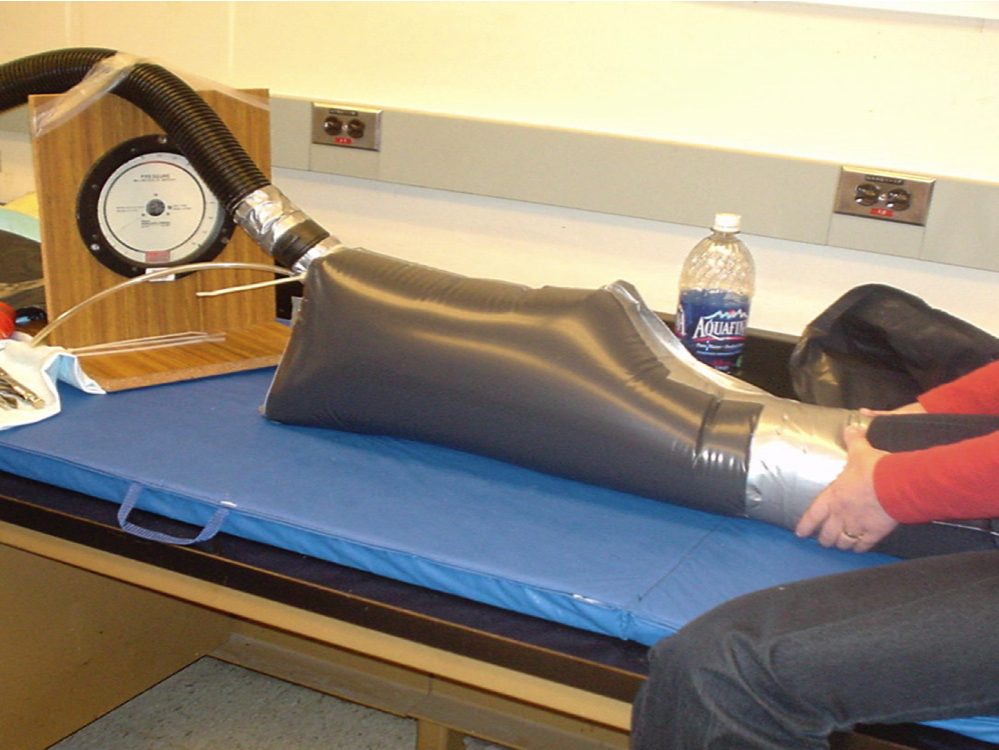
Lower extremity placed within inflatable pressure chamber up to the distal third of the thigh.

After the patients rested for 5 min, measurements were recorded using the custom‐made PPG device, which is a validated, noninvasive optical technique that measures local microvascular blood flow within skin and muscle (Morris and Woodcock 2002; Mateus and Hargens 2012). Prior to each measurement, the PPG device was calibrated by adjusting the intensity of each LED until a good signal‐to‐noise ratio was acquired (Mateus and Hargens 2012). Following this the PPG probe was positioned over the middle third of the anterior compartment of the leg and positioned 2–3 cm below the anterior tuberosity and 2–3 cm lateral to the anterior tibial crest. The probe was then loosely covered with a bandage to shield the probe from external light. Then, the leg was placed within a pressure chamber up to the distal third of the thigh (Fig. 1). The enclosure was sealed at this level by a wide blood pressure cuff inflated to 30 mmHg. Afterward, the enclosure was pressurized by compressed air at 30 mmHg that was measured with a digital pressure monitor (Model PM015D; World Precision Instruments Inc, Sarasota, FL). During the first 3 min of pressurization, steady‐state values for microvascular flows within skin and muscle were obtained. After the initial 3 min, measurements were recorded every 30 sec for 30 min. Within 5 min after mild pneumatic compression was stopped and the chamber removed, sensation tests were repeated.

Based on our study design and preliminary data obtained at the outset of this study, it was established that the variables of interest were not normally distributed. Thus, data were analyzed using a nonparametric Wilcoxon test with the Statistical Package for the Social Sciences (SPSS Inc, Chicago, IL). Statistical significance was established with *α *< 0.05. The null hypothesis showed no difference between baseline and pressurized values of sensation and microvascular blood flow in both healthy and diabetic patients.

## Results

All patients tolerated the experiment well and were able to finish the protocol. There were no reports of adverse events during or after the collection of our data. Patients denied any discomfort associated with application of compression by either the inflation air chamber or proximal sealing cuff.

In the healthy controls, when comparing measurements before and after mild continuous pneumatic compression, SBF increased by 14.9% (*N* = 19, SE = 7.47%, *P* = 0.12) and MBF increased significantly by 39.8% (*N* = 19, SE = 8.58%, *P* < 0.01). The diabetic group also showed a trend to higher SBF of 3.19% (*N* = 16, SE = 11.5%); however, this was also not statistically significant (*P* = 0.96). In this same group, MBF increased significantly by 17.7% (*N* = 16, SE = 5.39%, *P* = 0.03).

**Figure 2.**
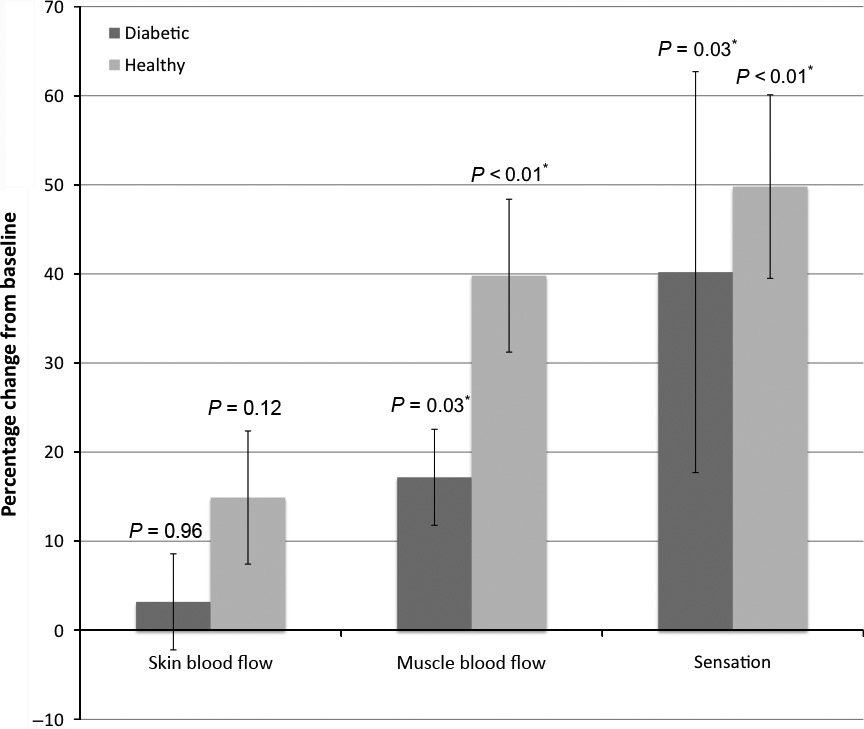
Changes in skin and muscle microvascular blood flows and foot sensation in diabetic patients (dark) and normal, healthy controls (gray). Error bars represent standard errors of the mean (±SE).

The threshold force for sensation, as determined by the Semmes‐Weinstein monofilaments, improved significantly in the healthy subjects by 49.8% (*N* = 14, SE = 10.3%, *P* < 0.01). The diabetic group also showed a significant improvement in sensation with a 40.2% increase (*N* = 13, SE = 22.5%, *P* = 0.03). These results are summarized in Table 2 and Figure 2. Overall, both the healthy control and diabetic groups had statistically significant increases in MBF and improvement in sensation.

**Table 2.** Percent change from baseline after application of 30‐mmHg pneumatic compression for a period of 30 min.

	Percent change from baseline (%)	*P* value*
Skin blood flow, Group I control	14.9 ± 32.6	0.12
Skin blood flow, Group II diabetics	3.19 ± 46.1	0.96
Muscle blood flow, Group I control	39.8 ± 37.4	<0.01
Muscle blood flow, Group II diabetics	17.7 ± 21.6	0.03
Sensory threshold, Group I control*	49.8 ± 38.4	<0.01
Sensory threshold, Group II diabetics*	40.2 ± 80.9	0.03

* Significant at *P* < 0.05.

* Sensation tested in first web space of foot using Semmes‐Weinstein monofilaments.

## Discussion

During this project, subjects underwent pressurization of the lower limb with mild, continuous pneumatic compression that significantly increased MBF and improved sensation in both groups. Previous reports have documented increased MBF during or after compression with elastic sleeves or IPC of the forearm or lower extremity (Eze et al. 1996; Delis 2005; Labropoulos et al. 2005). However, to our knowledge, this is the first study to document an increase of microvascular blood flow during continuous mild pneumatic compression, as well as improved sensation after pressurization. Moreover, these novel and counterintuitive results occur in both diabetic patients and healthy subjects, representing another novel finding of this study.

In both groups MBF and sensation improved. Therefore, the increase in muscle microvascular flow most likely is due to a myogenic response and/or increase in the arteriovenous gradient. Reneman et al. (1980) showed that pneumatic compression of rabbit hind paw increases venous and intramuscular pressures, while decreasing transmural pressure and dilating arterioles. Also, the use of four‐layer external compression banding of the leg resulted in an increase in blood flow, either by a myogenic response or release of a vasodilator substance release (Mayrovitz and Larsen 1997). Previous research documents the benefits of IPC for the treatment of lower extremity vascular disease (van Bemmelen et al. 1994; Eze et al. 1996). In this study, the healthy control and diabetic groups increased MBF by 39.8% (*P* < 0.01) and by 17.7% (*P* = 0.03), respectively. Also, foot sensation improved significantly in the control group by 49.8% (*P* < 0.01), and in the diabetic group by 40.2% (*P* = 0.03). In previous studies, MBF has been increase after compression with IPC (Eze et al. 1996; Delis et al. 2000). In this study, microvascular blood flow is also increased during continuous compression. Moreover, our data support the use of continuous, mild external pneumatic compression at 30 mmHg over 30 min.

Muscle microvascular blood flows increase in both healthy volunteers and diabetic patients during continuous, mild pneumatic compression. This response may be attributed to a myogenic response by which arteriolar dilation increases blood flow in a mildly compressed limb (Bochmann et al. 2005). This temporary improvement of peripheral microcirculation may have direct clinical implications to aid healing of lower extremity venous or ischemic ulcers in diabetic and nondiabetic patients. Importantly, the increase in blood flow can aid in the delivery of drugs to underperfused areas. It also maybe due to the vasodilatation the increase in permeability could allow larger size drugs to be delivered to these tissues.

Perhaps the most notable functional outcome of our results is significantly improved sensation resulting from mild, continuous pneumatic compression. Maintenance of sensation is critical in limb salvage and amputation prevention in diabetics. In this study, even though we had a temporary increase in foot sensation, the results suggest that neurologic function can be enhanced with mild and continuous, low‐level compression. Our present results are counterintuitive because loss of foot sensation and compartment syndromes may occur with pressures over 30 mmHg over a period of 8 or more hours. However, there may be a different physiologic response to externally applied compression as opposed to elevated internal muscle pressure associated with compartment syndromes.

There are some limitations to this study. For example, the control group is not perfectly matched for age. Diabetes, specifically type 2, is often a disease encountered with older age. The differences in magnitude of improved blood flow and sensation may be due to the fact that the control group is younger, with less vascular pathology and more physiologically fit than the diabetic group. In addition, only one pressurization level and exposure time were used throughout this project, namely, 30 mmHg and 30 min, respectively. There may be other pressures and exposure times that may produce an even greater improvement in blood flow and sensation. Furthermore, at the time of enrollment, all patients enrolled had intact skin and none had diabetic foot ulcers. Also, measurements recording were not performed for a long period of time; hence, we cannot assess the durability of this response. Lastly, we did not study the contralateral untreated lower limb response, so we cannot determine if this is a systemic or a local response. Skin temperature was not measured during pressurization and we cannot determine the role it played during our intervention.

There are some limitations related to the PPG hardware. For example, PPG data are expressed in relative terms, not absolute microvascular flows. Reliable PPG data are presently expressed with reference to some baseline condition. In essence we can take a measurement, provide a stimulus, and then take a subsequent measurement during or after the stimulus, and compare how the PPG signal changed from the reference condition. However, the technique is noninvasive and well validated (Zhang et al. 2001a,b; Mateus and Hargens 2012).

In order to obtain better and longer results, different pressures and times of pressurization should be assessed. We propose that mild, continuous pneumatic compression could be used as a nonoperative adjuvant treatment for patients suffering from microvascular disease and impaired perfusion that results in compromised tissue viability in the leg. In addition, mild, continuous, external pneumatic compression may improve treatment of diabetic foot ulcers in conjunction with other therapies.

## Acknowledgments

We thank Jaime Mateus, Ph.D., and the University of California San Diego Physics Electronics Shop for assisting in the development of a photoplethysmography device with greater accuracy and reliability.

## Conflict of Interest

Each author certifies that he has no commercial associations that might pose a conflict of interest in connection with the submitted article.
